# Editorial: Soft robotics based on liquid crystal elastomers (LCEs)

**DOI:** 10.3389/frobt.2022.1018819

**Published:** 2022-10-13

**Authors:** Zhijian Wang, Jennifer Boothby, Devin J. Roach, Qiguang He

**Affiliations:** ^1^ Key Laboratory of Aerospace Advanced Materials and Performance, Ministry of Education, School of Materials Science and Engineering, Beihang University, Beijing, China; ^2^ Applied Physics Laboratory, Johns Hopkins University, Laurel, MD, United States; ^3^ Sandia National Laboratories, Advanced Materials Laboratory, Albuquerque, NM, United States; ^4^ Mechanical Engineering and Applied Mechanics Department, University of Pennsylvania, Philadelphia, PA, United States

**Keywords:** liquid crystal elastomers, soft robots, soft actuators, liquid crystal, actuator

Soft robots have become a rapidly growing research field with a number of encouraging characteristics compared to their rigid counterparts, such as large deformation, flexibility, high degree of freedom, friendly human-machine interaction, and environmental adaptability. Under this context, versatile actuating materials and deformable structures have been widely developed in the designs and fabrications of soft machines. Liquid crystal elastomers (LCEs) are one type of soft actuating material, which integrates an elastic polymer network and anisotropic liquid crystal mesogens. LCE based actuators exhibit appealing actuation performance (large actuation strain, high work density, multiple actuation modes) and anisotropic thermal and mechanical properties. A variety of external stimuli can be adopted to trigger the actuation of LCE, including heat, light, chemical, electrical, and pressure. With the development of advanced manufacturing techniques, the LCE-based actuators can be fabricated via 3D printing (Roach et al., 2022; Wang et al., 2022) and electrospinning (He et al., 2021), offering more opportunities in a variety of length scales.

This Research Topic focuses on the recent efforts in LCEs and their applications, covering the design and fabrication of LCE-based actuators and robotic systems as well as the fundamental understanding of the thermomechanical properties of LCE-based structures. This topic accepted four research articles after the peer review process.

There are two research articles focusing on the design and fabrication of LCE-based soft robots. Song et al. demonstrated a simple approach to fabricate an LCE inchworm using the direct ink write (DIW) additive manufacturing technique. To enable the directional movement of the inchworm robot, silicone feet with specific microstructures are employed to provide anisotropic friction. Driven by electricity, this inchworm robot can crawl with a moving speed of 4.3 mm/min. This work shows that the application of DIW technique in printable responsive materials may contribute to achieve functional and integrated soft robotics with compact design. Minori et al. present a strategy for the design of a soft jumping mechanism based on the snap-through instability to amplify the power output. This robot comprises three components: An LCE actuator, an elastic shell and a latch. When the LCE is heated and contracted, the elastic shell can be inverted. Once the latch is triggered, the shell exhibits a snap-back transition to release the stored energy, causing the robot to jump. This robot exhibits a high specific power (26.4 W/kg), large power amplification ratio (8,000), and high energy efficiency (>60%), opening an avenue for soft robotic systems capable of rapid, high energy motion.

There are two fundamental theoretical articles in this topic. Cheng et al. propose a method to stabilize a light responsive LCE inverted pendulum utilizing the self-excited oscillation. This work is mainly based on the nonlinear dynamic theory of LCE and numerical simulations. The inverted pendulum system only possesses a static state or a self-stabilized oscillation state, the amplitude and frequency of the oscillations are determined by the system parameters such as light intensity and contraction ratio. This work produces a new understanding of inverted pendulum systems and their potential applications in the soft robotic field.

In addition to the promising actuation performance of the LCE, the anisotropic mechanical properties of the LCE can also be considered in developing novel structures. Liang et al. demonstrate the programmable mechanical metamaterials using nematic LCEs. Compared to conventional mechanical materials, the soft elasticity of nematic LCEs add additional variables for programing the stress-strain relation under uniaxial tension. This LCE-based metamaterial undergoes a transition between strain-softening and strain-stiffening states, enablingtunability of the mechanical properties. This work builds a framework for material-based programmability, representing substantial impact to the design of robotic systems.

The research articles collected in this special issue explore the applications of LCEs in the field soft robots and structures, from the mechanical design, theoretical modelling, and manufacturing. These works provide an insightful understanding in the development of LCE-based soft robots and structures.
